# Desafíos teóricos y prácticos del universalismo proporcional: una revisión^*^

**DOI:** 10.26633/RPSP.2021.102

**Published:** 2021-10-18

**Authors:** Florence Francis-Oliviero, Linda Cambon, Jérôme Wittwer, Michael Marmot, François Alla

**Affiliations:** 1 University of Bordeaux Bordeaux Francia University of Bordeaux, Bordeaux, Francia.; 2 Institute of Health Equity at the University College London Londres Reino Unido Institute of Health Equity at the University College London, Londres, Reino Unido.

**Keywords:** Equidad en salud, política de salud, factores socioeconómicos, Health equity, health policy, socioeconomic factors, Equidade em saúde, política de saúde, fatores socioeconômicos

## Abstract

**Objetivo.:**

En el 2010 se propuso el principio del universalismo proporcional como solución para reducir las desigualdades en materia de salud. Aunque tuvo una gran resonancia, no parece haber sido aplicado ampliamente y no existen directrices sobre cómo aplicarlo. Los dos objetivos específicos de esta revisión sistemática exploratoria fueron: 1) describir el contexto teórico en el que se estableció el universalismo proporcional, y 2) describir cómo los investigadores aplican el universalismo proporcional y las cuestiones metodológicas relacionadas.

**Métodos.:**

Se buscó en todas las bases de datos de la *Web of Science* los artículos publicados hasta el 6 de febrero del 2020 que tuvieran como tema “universalismo proporcional” o sus sinónimos “universalismo dirigido” o “universalismo progresivo”.

**Resultados.:**

Esta revisión de 55 artículos permitió obtener una visión global del universalismo proporcional en cuanto a sus fundamentos teóricos y su aplicación práctica. El principio del universalismo proporcional se basa en las teorías sociales del universalismo y el direccionamiento, y propone vincular estos dos aspectos para lograr una reducción efectiva de las desigualdades en materia de salud. Respecto de su aplicación práctica, las intervenciones basadas en este principio son poco frecuentes y dan lugar a diferentes interpretaciones. Todavía existen muchos desafíos metodológicos y éticos en relación con la concepción y evaluación de las intervenciones relacionadas con el universalismo proporcional, incluida la forma de aplicar la proporcionalidad y la identificación de las necesidades.

**Conclusión.:**

En esta revisión se llevó a cabo un mapeo de la literatura científica disponible sobre el universalismo proporcional y sus conceptos relacionados. Este principio se basa en teorías sociales. Tal como lo destacaron autores que implementaron intervenciones de universalismo proporcional, su aplicación plantea muchos desafíos, desde el diseño hasta la evaluación. El análisis de las aplicaciones del universalismo proporcional presentado en esta revisión respondió a algunos de ellos, pero los desafíos metodológicos restantes requieren ser abordados en futuras investigaciones.

## Abstract (en)

**Objective.:**

In 2010, the principle of proportionate universalism (PU) has been proposed as a solution to reduce health inequalities. It had a great resonance but does not seem to have been widely applied and no guidelines exist on how to implement it. The two specific objectives of this scoping review were: (1) to describe the theoretical context in which PU was established, (2) to describe how researchers apply PU and related methodological issues.

**Methods.:**

We searched for all articles published until 6th of February 2020, mentioning “Proportionate Universalism” or its synonyms “Targeted universalism” OR “Progressive Universalism” as a topic in all Web of Science databases.

**Results.:**

This review of 55 articles allowed us a global vision around the question of PU regarding its theoretical foundations and practical implementation. PU principle is rooted in the social theories of universalism and targeting. It proposes to link these two aspects in order to achieve an effective reduction of health inequalities. Regarding practical implementation, PU interventions were rare and led to different interpretations. There are still many methodological and ethical challenges regarding conception and evaluation of PU interventions, including how to apply proportionality, and identification of needs.

**Conclusion.:**

This review mapped available scientific literature on PU and its related concepts. PU principle originates from social theories. As highlighted by authors who implemented PU interventions, application raises many challenges from design to evaluation. Analysis of PU applications provided in this review answered to some of them but remaining methodological challenges could be addressed in further research.

## RESUMO

**Objetivo.:**

Em 2010, o princípio do Universalismo Proporcional (UP) foi proposto como uma solução para reduzir as desigualdades na saúde. Houve uma grande receptividade, mas o princípio parece não ter sido amplamente aplicado e não há diretrizes sobre como implementá-lo. Os dois objetivos específicos desta análise de escopo foram: (1) descrever o contexto teórico no qual o UP foi estabelecido e (2) descrever como os pesquisadores aplicam o UP e questões metodológicas correlatas.

**Métodos.:**

Buscamos em todas as bases de dados científicas da Web todos os artigos publicados até 6 de fevereiro de 2020 que mencionavam, como tema, o “Universalismo Proporcional” ou seus sinônimos “Universalismo Visado” **ou** “Universalismo Progressivo”.

**Resultados.:**

Essa análise de 55 artigos nos permitiu ter uma visão global relacionada com o UP, suas bases teóricas e sua implementação na prática. Os princípios do UP têm suas raízes nas teorias sociais do universalismo e na definição de metas. A proposta é vincular esses dois aspectos para atingir uma redução efetiva das desigualdades em saúde. Com relação ao aspecto prático da implementação, as intervenções de UP foram raras e levaram a diferentes interpretações. Ainda há muitos obstáculos metodológicos e éticos relativos ao conceito e à avaliação das intervenções de UP, inclusive sobre como aplicar o aspecto da proporcionalidade e a identificação das necessidades.

**Conclusões.:**

Esta análise fez um mapeamento da literatura científica disponível sobre UP e seus conceitos correlatos. O princípio por trás do UP tem suas origens nas teorias sociais. Como destacam os autores que implementaram as intervenções de UP, sua aplicação produz muitas dificuldades, da elaboração à avaliação. A análise das aplicações de UP fornecidas nesta pesquisa permitiu a obtenção de algumas respostas. No entanto, pesquisas futuras poderiam abordar as dificuldades metodológicas remanescentes.

Las desigualdades en la salud son un problema ubicuo y  cada vez mayor en todo el mundo, muy a menudo descrito mediante el gradiente social de la salud: independientemente del indicador de privación (ingresos, categoría social, etc.) considerado, cuanto más pertenezca a las clases más desfavorecidas, peor será la salud de una persona (1). Estas desigualdades existen dentro de los países al igual que entre ellos. La Organización Panamericana de la Salud (OPS) proporciona el siguiente ejemplo, entre muchos: “la diferencia en la esperanza de  vida general fue de 19 años tanto para los hombres como para las mujeres en el 2016” (2) en los países de la Región de las Américas.

En el 2010, en la revisión *Fair Society, Healthy Lives* se propuso el principio del universalismo proporcional como una solución para reducir estas desigualdades (3). Las acciones deben ser universales, pero con una intensidad y una escala que sean proporcionales al nivel de la desventaja: esa es la definición exacta del universalismo proporcional. Poco después de su publicación, esta revisión tuvo una gran resonancia entre expertos de diferentes campos y hubo muchas reacciones alentadoras o contradictorias (4-6). Por otro lado, una búsqueda rápida de la palabra clave “universalismo proporcional” muestra que recientemente el principio ha cobrado impulso. A pesar de esta preocupación cada vez mayor entre los investigadores, a excepción de unas pocas políticas locales en Inglaterra y en los países europeos nórdicos, el principio no parece que se haya aplicado ampliamente.

Hasta donde sabemos, no hay pautas sobre cómo poner en práctica las políticas o intervenciones de salud que cumplen este principio ni sobre cómo evaluarlas. La comprensión de los referentes teóricos podría contribuir a desarrollar aplicaciones más eficientes y satisfactorias. Asimismo, el análisis de las intervenciones o los programas de salud que hacen referencia al universalismo proporcional podría poner de relieve algunas cuestiones prácticas.

El objetivo de la presente revisión sistemática exploratoria fue mapear la bibliografía disponible que se refería al concepto de universalismo proporcional o a sus conceptos conexos. Los dos objetivos específicos fueron: 1) describir el contexto teórico en el cual se estableció el universalismo proporcional, y 2) describir cómo los investigadores aplican el universalismo proporcional y las cuestiones metodológicas conexas.

## MATERIALES Y MÉTODOS

### Marco de la revisión

Dado el gran alcance de la cuestión investigada, su naturaleza emergente y la heterogeneidad de los artículos analizados, realizamos una revisión sistemática exploratoria del material publicado en conformidad con las directrices de PRISMA-ScR y el Instituto Joanna Briggs (JBI) (7,8). Seguimos los cinco pasos metodológicos obligatorios propuestos por Arksey y O’Malley y los completamos secundariamente: 1) determinar la cuestión objeto de investigación, 2) encontrar los estudios pertinentes, 3) seleccionar los estudios, 4) mapear los datos, 5) compilar, resumir y presentar los resultados (9-11).

### Fuentes de información y estrategia de búsqueda

Buscamos todo tipo de artículos publicados hasta el 6 de febrero del 2020 que mencionaban el “universalismo proporcional” o sus sinónimos, “universalismo dirigido” o “universalismo progresivo”, como tema en todas las bases de datos de la Web of Science escritas en inglés o francés. Utilizamos la Web of Science para poder encontrar documentos científicos de diferentes campos.

### Selección de los estudios

Cada título y resumen fueron examinados por dos autores (FF, FA), y las discrepancias fueron resueltas mediante la discusión. Incluimos artículos que definían, analizaban los detalles y describían las aplicaciones del universalismo proporcional o de sus conceptos conexos. La búsqueda se limitó al contexto de la salud pública o de las políticas sociales. Se excluyeron los artículos que no estaban disponibles o no estaban directamente relacionados con uno de los conceptos mencionados.

### Análisis de los artículos

Se usó una cuadrícula de lectura para analizar sistemáticamente los artículos, que contenía: 1) características de los artículos: autor, región del primer autor, tipo de artículo, campo; 2) elementos de definición para los términos clave; y 3) objetivo, métodos, principales resultados y preguntas planteadas por los autores con respecto a la implementación del universalismo proporcional. Los datos fueron analizados usando Excel.

## RESULTADOS

### Características de los artículos

**FIGURA 1.**
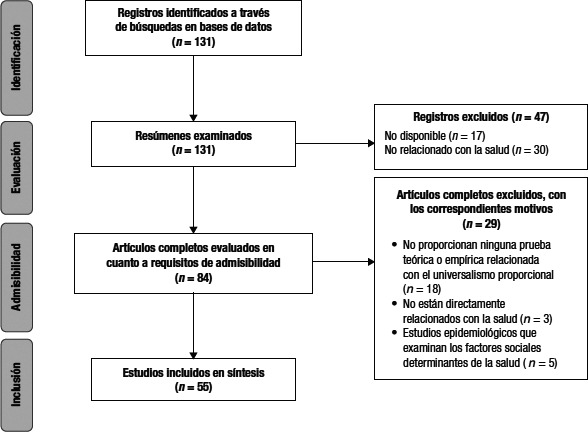
Selección de los artículos

Nuestra búsqueda inicial arrojó 131 artículos. Tras la selección, finalmente se incluyeron 55 artículos (figura 1), que fueron de diferentes tipos (intervenciones, artículos teóricos y revisiones).

**FIGURA 2.**
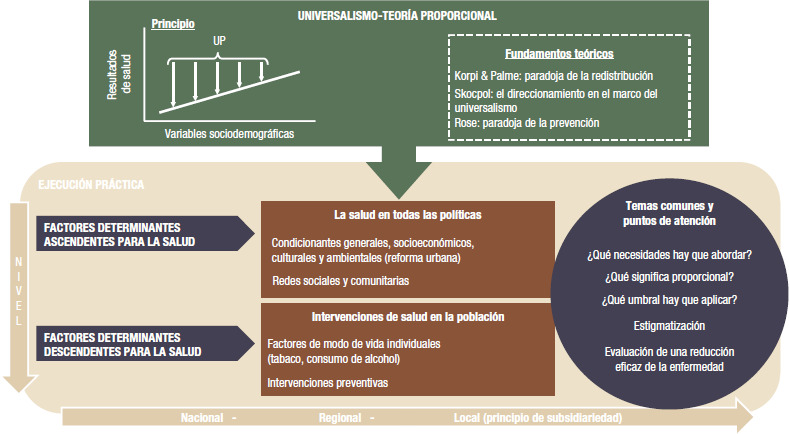
Resumen gráfico del examen

La mayoría de los estudios incluidos correspondieron a países europeos (n = 39), seguidos de países de América del Norte y del Sur (n = 9) (cuadro 1). Del total de artículos, 56,4% (n = 31)se publicaron a partir del 2016. Las características de las intervenciones en materia de salud de la población se describen en el cuadro 2. En la figura 2, se proporciona un resumen gráfico de la revisión: presenta el contexto teórico del universalismo proporcional y cuestiones comunes planteadas por las aplicaciones del universalismo proporcional orientadas a los factores determinantes ascendentes o descendentes.

### Contexto teórico subyacente al universalismo proporcional

Los conceptos teóricos relativos al universalismo proporcional se refieren a dos conceptos principales: el universalismo y el direccionamiento, ampliamente desarrollados en la bibliografía, especialmente en las ciencias humanas y sociales (cuadro 1). Si la noción de universalismo se remonta al siglo de la Ilustración en Europa, la oposición entre universalismo y direccionamiento como sistemas para reducir las desigualdades ha sido un tema de debate entre científicos sociales y políticos durante los últimos treinta años, y actualmente sigue siéndolo.

En un artículo fundamental, Korpi y Palme describieron la paradoja de la redistribución: una política universal es más redistributiva que una dirigida. Confrontaron clásicamente las dos: una política es uno o lo otro (12). No obstante, a veces la distinción entre ambos términos se diluye (13). Además, muchos investigadores han agregado matices, que en particular sugieren que, por un lado, deberíamos tener en cuenta los propósitos de las políticas y, por el otro, sus resultados. Por ejemplo, una política puede tener un propósito universal: una familia se beneficia con cada hijo nacido. Sin embargo, si las familias con ingresos más bajos concentran a las que tienen un mayor número de hijos, entonces esa política parece orientarse a sus resultados (por ejemplo, una familia con más hijos recibirá más dinero) (14-16). Por consiguiente, estos investigadores propugnan definir las políticas con arreglo a sus propósitos y no a sus resultados (16).

**CUADRO 1.** Características de los artículos incluidos (*n*= 55)

	Campo	País	Año de publicación
**Ciencias social.es, económicas y políticas**			
Brady et al. (21)	Universalismo/direccionamiento	Alemania	2012
Brady et Bostic (28)	Universalismo/direccionamiento	Alemania/Irlanda	2015
Carey et al. (39)	Universalismo/direccionamiento	Australia	2015
Carey et al. (41)	Universalismo/direccionamiento	Australia	2016
Carey et al. (18)	Universalismo/direccionamiento	Australia	2017
Fischer (20)	Universalismo/direccionamiento	Reino Unido	2010
Grogan et al. (13)	Universalismo/direccionamiento	Estados Unidos	2003
Horton et al. (32)	Universalismo/direccionamiento	Reino Unido	2010
Imai (23)	Universalismo/direccionamiento	India	2007
Jacques (16)	Universalismo/direccionamiento	Canadá	2018
Kabeer (29)	Universalismo/direccionamiento	Reino Unido	2014
Kim (25)	Universalismo/direccionamiento	Japón	2010
Kuival.ainen et al. (22)	Universalismo/direccionamiento	Finlandia	2010
Marchal. et al. (15)	Universalismo/direccionamiento	Bélgica	2019
Skocpol (33)	Universalismo/direccionamiento	Estados Unidos	1991
Corburn (65)	Salud en todas las políticas	Estados Unidos	2014
Brewster et al. (53)	Salud dental	Reino Unido	2013
Briançon et al. (64)	Obesidad infantil	Francia	2020
Dierckx et al. (57)	Salud maternoinfantil	Bélgica	2019
Dodge et al. (40)	Salud maternoinfantil	Estados Unidos	2019
Cruz-Martinez et al. (17)	Pensiones para la vejez	América Latina y el Caribe	2019
Moffatt et al. (67)	Pensiones para la vejez	Reino Unido	2007
Müller (24)	Pensiones para la vejez	Bolivia	2009
Neelsen et al. (30)	Cobertura de salud específica	Perú	2017
Van Lancker et al. (15)	Pobreza de madres solteras	Europa	2015
Van Lancker et al. (14)	Pobreza infantil	Europa	2015
Van Vliet J (41)	Ninguno en particular	Suecia	2018
Benach et al. (46)	Ninguno en particular	España	2012
Porcherie et al. (43)	Ninguno en particular	Francia	2017
Sannino et al. (68)	Ninguno en particular	Francia	2018
**Salud pública, epidemiología**			
Barboza et al. (62)	Salud maternoinfantil	Suecia	2018
Burström et al. (37)	Salud maternoinfantil	Suecia	2017
Barlow et al. (61)	Salud maternoinfantil	Reino Unido	2010
Bywater et al. (52)	Salud maternoinfantil	Reino Unido	2018
Hogg et al. (73)	Salud maternoinfantil	Reino Unido	2013
Thomson et al. (35)	Salud maternoinfantil	Reino Unido	2012
Cowley et al. (54)	Salud maternoinfantil	Reino Unido	2014
Morrison et al. (56)	Salud maternoinfantil	Reino Unido	2014
Maharaj et al. (47)	Salud maternoinfantil	Reino Unido	2012
Egan et al. (49)	Reforma urbana	Reino Unido	2016
Guillaume et al. (38)	Detección del cáncer	Francia	2017
Guillaume et al. (51)	Detección del cáncer	Francia	2017
Legrand et al. (50)	Obesidad infantil	Francia	2017
Mc Laren (31)	Universalismo/direccionamiento	Canadá	2019
Rice P (39)	Prevención de alcoholismo	Reino Unido	2019
Vitus et al. (70)	Control de peso corporal	Dinamarca	2017
Welsh et al. (55)	Salud mental, pediatría	Australia	2015
Bekken et al. (74)	Ninguno en particular	Noruega	2018
Affeltranger et al. (44)	Ninguno en particular	Francia	2018
Goldblatt P (59)	Ninguno en particular	Reino Unido	2016
Wiseman et al. (75)	Cobertura universal de salud	Indonesia	2018
**Ética**			
Darquy et al. (48)	Detección del cáncer	Francia	2018
Lechopier et al. (72)	Detección del cáncer	Francia	2017
Moutel et al. (71)	Detección del cáncer	Francia	2019
Devereux S (63)	Ninguno en particular	Reino Unido	2016

**CUADRO 2.** Descripción de las intervenciones en materia de salud de la población (n = 9)

Autor, año	Principio de referencia	Objetivos fijados en el artículo	Descripción de la intervención	Evaluación del “nivel de desventaja”	Evaluación de la reducción de desigualdad	Principales hallazgos
Hogg et al. 2012	Universalismo proporcional	Examinar la evaluación de la vulnerabilidad y las necesidades de apoyo de las familias, desde la perspectiva de los padres y los visitadores de salud, con un enfoque particular en el Lothian Child Concern Model.	A cada familia se le ofrecen cuatro visitas domiciliarias del visitador de salud entre 10 días y 4-6 meses después del parto, durante las cuales los padres y el visitador de salud hablan sobre la salud y las necesidades sanitarias de la familia, y ese último proporciona información y consejo según sea necesario.	Individual, a través de visitas domiciliarias	Estudio cualitativo (entrevistas de los padres y los visitadores de salud)	Un hallazgo significativo del estudio es el concepto de “universalismo progresivo”, que proporciona un apoyo continuo a las familias en función de sus necesidades. Las madres desearían más colaboración con los visitadores de salud.
Maharaj et al. 2012	Universalismo proporcional	1) Demostrar que la pediatría comunitaria puede contribuir a reducir las desigualdades de la salud proporcionando servicios que sean accesibles para los niños y que preferentemente usen aquellos niños cuya salud sea probable que resulte afectada por la privación. 2) Proporcionar un modelo para que otros puedan mejorar y vigilar la equidad en los servicios que prestan.	Nueva organización del servicio de pediatría. Las principales funciones del nuevo modelo de servicio son la colaboración entre organismos, la accesibilidad, la evaluación holística, la prestación exhaustiva de servicios y el hecho de que el servicio esté disponible para todos y pueda atender proporcionalmente a los niños con mayores niveles de necesidad.	Índices de privación múltiple	Acceso a la atención en el campo de intervención en comparación con un campo similar, descrito por quintil de privación	La nueva tasa de contacto de pacientes para los niños más desfavorecidos de la población fue más de tres veces superior a la correspondiente a los menos desfavorecidos [riesgo relativo aproximado (OR) 3,29, intervalo de confianza (CI) de 95%, 2,76-3,93].
Guillaume et al. 2017	Universalismo proporcional	Evaluar la eficacia de la mamografía móvil pare reducir las desigualdades sociales y geográficas con respecto a la participación en la detección del cáncer de mama en una población general bien definida de un territorio francés.	Detección nacional del cáncer de mama combinada con mamografía móvil en un departamento rural en Francia (Orne). Ensayo en un departamento rural francés.	Sin especificar	Sin especificar	Después del ajuste, la invitación se asoció con un aumento significativo de la participación individual (riesgo relativo aproximado =2,9)
Legrand et al. 2017 Briançon et al. 2020	Universalismo proporcional	Evaluar la eficacia de una intervención basada en la escuela para abordar las desigualdades sociales en adolescentes con sobrepeso y el impacto de las intervenciones en la adopción de comportamientos saludables, la calidad de vida, la ansiedad y la depresión.	Se propuso una gestión sanitaria estándar para todos los adolescentes de conformidad con el ensayo PRALIMAP, y una gestión sanitaria fortalecida que abordara las limitaciones solamente para los adolescentes socialmente menos favorecidos del grupo objeto de la intervención.	Puntuación en la escala de bienestar familiar	Comparación del IMC después de la intervención entre tres grupos establecidos con los resultados en la puntuación de la escala de bienestar familiar.	Tendencia a obtener valores más altos para el grupo menos favorecido al que se ofreció una gestión sanitaria fortalecida (reducción del IMC -0,06 [-0,11 a -0,01]
Burstrom et al. 2017 Barboza et al. 2018	Universalismo proporcional	Se esperaba que la intervención fortaleciera el conocimiento de los padres sobre sus hijos, mejorara la interacción entre padres e hijos, aumentara los contactos de los padres con otros actores pertinentes de la sociedad y reforzara su bienestar y su eficiencia personal.	Programa ampliado de visitas posnatales domiciliarias: la intervención consistía en cinco visitas domiciliarias suplementarias cuando el niño tenía entre 2 y 15 meses de edad, realizadas conjuntamente por una enfermera pediátrica y un asesor parental del servicio social, ofrecidas a todos los padres de primeros hijos que acudieron al centro de salud infantil de Rinkeby.	Se ofrecen cinco visitas domiciliarias suplementarias a todos los padres. Los padres expresan su deseo de acudir a visitas suplementarias.	Enfoques de métodos combinados (entrevistas, tasas de participación, examen de los expedientes infantiles...)	Protocolo del estudio
Autor, año	Principio de referencia	Objetivos fijados en el artículo	Descripción de la intervención	Evaluación del “nivel de desventaja”	Evaluación de la reducción de desigualdad	Principales hallazgos
Bywater et al. 2018	Universalismo proporcional	¿Las fases de E-SEE mejoran el bienestar social y emocional infantil a los 20 meses de edad si se compara con los servicios prestados de forma habitual?	Todos los padres de la intervención recibirán un libro infantil titulado Incredible Years [años maravillosos] (a nivel universal) y se les puede ofrecer programas basados en grupos de lactantes o bebés basándose en la valoración de la depresión de los progenitores obtenida en el cuestionario de salud del paciente o las evaluaciones del bienestar social y emocional infantil obtenidas del cuestionario sobre edades y etapas: Social Emotional (segunda edición; ASO:SE-2). Los padres del grupo de control recibirán los servicios habituales.	Cuestionario de salud del paciente o valoraciones del bienestar social y emocional infantil del cuestionario sobre edades y etapas: Social Emotional (segunda edición; ASO:SE-2).	Sin especificar	Protocolo del estudio
Darquy et al. 2018	Universalismo proporcional	Abordar las desigualdades socioeconómicas halladas en los programas existentes para la detección del cáncer, en consonancia con las prioridades establecidas en el plan nacional francés contra el cáncer 2014-2019.	Un programa abierto a todas las mujeres de 25 a 65 años de edad, con intervenciones orientadas a poblaciones examinadas identificadas (mujeres mayores de 50 años que no son conscientes de sus riesgos, mujeres en situación precaria u homosexuales, poblaciones vulnerables, mujeres que corren mayor riesgo de cáncer cervicouterino).	Estudios preliminares para cuantificar las mujeres que no participan en los programas de detección universal sistemática.	Sin especificar	Protocolo del estudio
Dodge et al. 2019	Universalismo dirigido	Descripción y evaluación del programa “Family Connects”.	Modelo de conexión familiar, basado en tres pilares: una o más visitas domiciliarias (tras el nacimiento del hijo), armonización comunitaria (recursos comunitarios disponibles para las familias) y datos y vigilancia (registro sanitario electrónico compartido por todas las partes interesadas).	Individual, a través de visitas domiciliarias	Dos ECA (ensayos clínicos controlados aleatorizados) y un ensayo sobre el terreno (entrevistas de padres): conectividad, crianza y salud mental de los padres, salud y bienestar infantil.	Las familias de los grupos objeto de las intervenciones comunicaron más conexión con los recursos comunitarios, comportamientos de crianza más positivos y menos lesiones o enfermedades graves entre sus hijos.

El direccionamiento es una noción compleja. Cruz-Martínez distinguió diferentes formas de direccionamiento: por los medios o por la categoría social (17). En su glosario, Carey define diferentes formas de direccionamiento, que denominó selectivismo y particularismo negativos y positivos. El selectivismo negativo podría considerarse como una comprobación de los medios económicos, que corresponde a la medida de los ingresos de las personas para decidir si tienen derecho a asistencia social. El selectivismo positivo se refiere al direccionamiento basado en las necesidades, independientemente de la posición social; mientras que el particularismo propone diferentes criterios para distintas categorías que reflejan circunstancias diversas (18).

Noy deploraba el hecho de que direccionamiento a menudo se confunde con la comprobación de medios económicos, lo que puede dar lugar a interpretaciones erróneas (19). Se trata de dos conceptos relacionados, aunque distintos. El direccionamiento implica centrarse en un “segmento particular de la población” (estado característico, ubicación…), mientras que la comprobación de los medios económicos solo se centra en los ingresos. Según la autora, esta distinción es importante porque algunas críticas del direccionamiento son en realidad críticas de la rigurosidad del cumplimiento de la comprobación de los medios económicos, utilizado para asegurar que solo reciben prestaciones los destinatarios previstos (19).

Muchos expertos han analizado las ventajas y desventajas del direccionamiento y el universalismo (15-17, 20-30). A pesar de ser un principio aparentemente costo-eficaz, el direccionamiento da lugar a muchos problemas: estigmatización, mayor distancia social entre los beneficiarios y no beneficiarios, gasto administrativo de la comprobación de medios económicos, y también a clasificaciones erróneas, como infracoberturas y pérdidas (20,31,32). Por su parte, los partidarios del direccionamiento argumentan que los enfoques universales aumentan las desigualdades y conllevan costos significativos para la sociedad.

Con posterioridad a estas controversias, en 1991 Théda Skocpol propuso el “direccionamiento en el marco del universalismo”, que combina ambos enfoques, a veces llamado “universalismo progresivo”. Propuso unos marcos universales de política para ofrecer prestaciones y servicios suplementarios que ayuden desproporcionadamente a las personas menos privilegiadas sin estigmatizarlas (33). En 1985, Rose introdujo este debate en la política de salud pública (34.31). Según él, los riesgos para la salud se distribuyen entre un “continuo de riesgo”. Por lo tanto, describió la paradoja de la prevención como una medida preventiva que aporta grandes beneficios a la comunidad, pero poco a cada persona participante. Como las causas de muchas enfermedades se relacionan con factores sociales determinantes de la salud, Rose argumentó que la medicina y la política no deben separarse. De manera análoga a los profesionales de las ciencias sociales, en su libro expuso las ventajas y desventajas de las estrategias de prevención a nivel de la población general frente a las de alto riesgo (2,3).

Unos años después, la revisión *Fair Society, Healthy Lives* introdujo el principio de universalismo proporcional, una forma de abordar la dicotomización entre universalismo y direccionamiento en el campo de la salud (3). Basado en la teoría de los determinantes sociales de la salud, el enfoque del universalismo proporcional propone centrarse en los factores determinantes ascendentes, y desde este punto de vista propugnar políticas sociales centradas, por ejemplo, en la educación o el empleo.

### Diseño de intervenciones y políticas de universalismo proporcional: retos metodológicos

A pesar de esta abundante bibliografía, siguen existiendo lagunas que hay que abordar al diseñar programas y políticas basadas en estos principios (18,35). Para ilustrar estas cuestiones, en el cuadro 3 se brindan ejemplos de universalismo dirigido y una aplicación del universalismo proporcional. Ambos podrían contribuir a alcanzar el Objetivo de Desarrollo Sostenible 3.4, “para 2030, reducir en un tercio la mortalidad prematura por enfermedades no transmisibles” mediante la reducción de la prevalencia del sobrepeso y la obesidad (36).

### Contexto y nivel de las intervenciones

El material publicado mostró diferentes aplicaciones e interpretaciones del principio de universalismo proporcional, en particular con respecto a la variedad de áreas temáticas abordadas: detección del cáncer (n=5), salud maternoinfantil (n=9), salud y pobreza infantil (n=4), etc. (cuadro 1).

En la bibliografía, el nivel de las intervenciones en las que el universalismo proporcional puede aplicarse como un principio también fue heterogéneo. Diferentes autores lo proponen como un principio para concebir políticas de salud pública a nivel nacional o regional, como las políticas de prevención del alcoholismo y de detección del cáncer, o intervenciones más personalizadas, como los programas de visitas domiciliarias (37–41). Carey et al. propusieron un marco basado en el principio de subsidiariedad, que argumenta que, para aplanar el gradiente social, deben producirse escalas combinadas de intervenciones (42). Esta autora insiste en el hecho de que todas las intervenciones de salud deberían integrarse en un panorama más amplio para alcanzar tales objetivos. Se trata de una visión sistémica del universalismo proporcional, que también propusieron Porcherie et al. (43). Sea cual sea el nivel de la acción, los programas nacionales deben ser coherentes e interactuar con los de ámbito local (28,44). Los diferentes niveles de ejecución del universalismo proporcional (micro, meso o macro) no son mutuamente excluyentes, y si se combinan pueden esperarse resultados más eficientes (45).

**CUADRO 3.** Ejemplos de universalismo proporcional y universalismo dirigido con respecto al logro del ODS 3.4 (ejemplo específico: reducción del sobrepeso y la obesidad)

	Programa La salud en todas las políticas de Richmond (65)	Estudio de PRALIMAP-INES (64)
**Nivel de aplicación**	Política local	Intervención de salud de alcance nacional
**Descripción general de la política e intervención**	Ejecución de seis áreas de intervención final de salud en todos los programas de políticas: gobernanza y liderazgo desarrollo económico y educación servicio integral y comunidades seguras entornos edificados del vecindario salud ambiental y justicia servicios sociales y de salud en los hogares accesibles y de calidad.	1) Determinación de la categoría social mediante una puntuación de privación. 2) Composición de tres grupos: un grupo de control (gestión ordinaria de la atención) para grupos socialmente favorecidos; dos grupos de intervención aleatorios entre adolescentes más desfavorecidos: un grupo de control y gestión ordinaria y fortalecida de la atención
**Impulsor de la acción**	Factores determinantes ascendentes	Factores determinantes descendentes
**Objetivo perseguido por los autores**	Promover el desarrollo de tiendas de comestibles sanos mediante la zonificación del cultivo de la tierra (un objetivo específico del tercer eje)	Evaluar la intervención basada en la escuela para abordar las desigualdades sociales en los adolescentes con sobrepeso y la repercusión de las intervenciones orientadas a adoptar comportamientos saludables, mejorar la calidad de vida, y reducir la ansiedad y depresión.
**Población destinataria**	Población de Richmond, especialmente las zonas menos favorecidas	Adolescentes de 35 escuelas públicas primarias y secundarias (noroeste de Francia)
**Parte universal de la política e intervención**	Metas generales de equidad en salud para la ciudad	Gestión ordinaria de la atención (cinco sesiones educativas colectivas)
**Parte específica de la política e intervención**	Poblaciones y lugares para ayudar a grupos específicos y vecindarios actualmente vulnerables a mejorar la salud	La gestión fortalecida de la atención con miras a abordar las limitaciones solo se propuso para los adolescentes socialmente más desfavorecidos: tres reuniones multidisciplinarias (médicos y enfermeras escolares, dietistas, psicólogos, etc.); combinación de diferentes actividades orientadas: talleres sobre alimentación, promoción de la salud entre compañeros, cupón de equipo deportivo (40 €), tratamiento hospitalario especializado de la obesidad, entrevistas de motivación de la actividad física y entrevistas de motivación.
**Evaluación del nivel de desventaja**	Mediante la identificación de factores impulsores clave de las desigualdades	Escala de prosperidad familiar (cinco categorías sociales)
**Criterios de evaluación del mejoramiento de la salud**	% dicen haber consumido frutas/vegetales 3+ de la última semana % adultos realizaron actividad física regular en la última semana % señalan tener mala salud (autonotificación)	Comparación del IMC después de la intervención entre tres grupos establecidos, según la puntuación de la escala de prosperidad familiar

IMC, índice de masa corporal.

### Objetivos previstos

Los objetivos de las intervenciones resultantes del universalismo proporcional deben ser mejorar la salud general de la población y reducir el gradiente social (46). En la mayoría de las intervenciones incluidas en la revisión, los autores señalaron que se proponían reducir las desigualdades en la salud y centrarse en las desigualdades de acceso y geográficas, pero no reducir directamente el gradiente social (cuadro 2) (37,38,47–53). Las revisiones incluidas tampoco determinaron las intervenciones que perseguían este objetivo (54–56). Welsh et al., en una revisión para determinar las intervenciones que promovían el bienestar o prevenían las enfermedades mentales, expusieron que muy pocas intervenciones fueron específicamente diseñadas para abordar las inequidades o evaluar el impacto diferencial (42).

### Diferentes interpretaciones del universalismo proporcional

La definición de universalismo proporcional podría dar lugar a diferentes interpretaciones en la práctica. Dierckx, en su evaluación de tres casos de centros de trabajo social infantil y familiar, puso de manifiesto que los profesionales sobre el terreno se mostraron en desacuerdo con la definición de universalismo proporcional (57).

Por otro lado, en la bibliografía la interpretación de la noción de intensidad proporcional de la acción de salud llevada a cabo es heterogénea. ¿Tiene que ser la misma intervención con diferentes intensidades, como una prestación social que aumenta a medida que se incrementan las necesidades? (17) ¿O distintas intervenciones para diferentes grupos destinatarios? (58,59) Como alternativa, ¿la noción de proporción debe entenderse en el contexto de una política que se aplique más a las categorías desfavorecidas de la población (por ejemplo, el precio unitario mínimo para las bebidas alcohólicas o los impuestos sobre las bebidas azucaradas)? (39,45) En efecto, tales intervenciones legislativas y regulatorias son universales por naturaleza, y como se ha demostrado que el consumo también sigue un gradiente, el efecto en consecuencia se ajustaría de forma natural. Esta pregunta se hace eco de la que plantearon los profesionales de las ciencias sociales con respecto a la diferencia entre los propósitos, los resultados inmediatos y los resultados intermedios de una política.

Benach et al. introdujeron categorías de “política universal con énfasis adicional en los déficits” y de “política redistributiva” como escenario cercano pero diferente del universalismo proporcional (60). El universalismo proporcional consiste en un enfoque en que los beneficios aumentan a lo largo del gradiente y se reduce la brecha entre los grupos socioeconómicos (60). En nuestra revisión, la mayoría de las intervenciones fueron visitas domiciliarias y, por lo tanto, constituyen por naturaleza universalismo proporcional porque son prácticamente atención individual, o bien una suma de intervenciones orientadas a los más desfavorecidos (45,61). Aunque fueran proporcionales, en su mayor parte no fueron universales sino específicamente ejecutadas en una zona desfavorecida (37,50,62).

Tras abordar la cuestión de la proporcionalidad, también surgió la cuestión de un umbral. En efecto, la intervención más proporcional sería, por su propia naturaleza, individual, lo cual no es posible realizar por limitaciones evidentes de viabilidad. Así pues, cuando se aborda el tipo de desigualdad, ¿qué umbral y qué nivel de detalle deben aplicarse a lo largo del gradiente? (18) Sin duda, incluso un direccionamiento proporcional implica establecer un “umbral de pobreza” justo por encima del cual las personas recibirán menos y pueden percibirlo como injusto (63). En algunas entrevistas, Thomson et al. cuestionaron las experiencias de las mujeres que se beneficiaron de los servicios de atención prenatal y observaron que las mujeres con embarazos de bajo riesgo percibían un cierto grado de inequidad en la prestación de dichos servicios (35).

Para adaptar las intervenciones de forma proporcional al nivel de desventaja o necesidad, es necesario determinar cuáles podrían ser las necesidades. Esta cuestión está relacionada con la de los factores determinantes ascendentes y descendentes que se proponen abordar las intervenciones (59). Entre las intervenciones incluidas en nuestra revisión, algunas se centraban en los factores determinantes ascendentes (reforma del vecindario, crianza), y otras en los descendentes (detección del cáncer, control de la obesidad) (50,64). Cuando los factores determinantes descendentes eran el impulsor de la acción, los autores se centraron más fácilmente en el acceso a la atención o el riesgo para la salud (48,51). En el caso de los factores determinantes ascendentes, la evaluación de las necesidades puede realizarse de acuerdo con los ingresos, el índice o nivel socioeconómico, la categoría social o la categoría territorial. Un ejemplo de abordar los factores determinantes ascendentes podría ser la incorporación de “la salud en todas las políticas”, lo que significa aplicar políticas no directamente orientadas a mejorar la salud, como las políticas ambientales o educativas, sino con efectos indirectos previstos sobre la salud. La ciudad de Richmond, en California, estableció un nuevo marco basado en este principio, en referencia al universalismo dirigido y la democracia participativa (cuadro 3) (65). Tras determinar las necesidades, el siguiente paso es conseguir que no se produzcan clasificaciones erróneas. Brewster et al. ensayaron diferentes técnicas de medición del riesgo para la salud a fin de determinar si una intervención dirigida alcanzaba su objetivo (53). Observaron que casi 50% de los niños seleccionados con arreglo a su código postal (selección de zonas más desfavorecidas) no estaban verdaderamente en situación de riesgo con respecto a otras mediciones (antecedentes clínicos, otros índices de privación) (53). Según Cornia y Stewart, pueden producirse dos tipos de errores de selección: exclusión, cuando una intervención no logra detectar a las personas necesitadas, e inclusión, cuando la intervención detecta a personas equivocadas (63,66). Otro punto de interés reside en el hecho de que las personas que tienen derecho a recibir algún tipo de ayuda no necesariamente la aprovechan, principalmente porque no saben que tienen derecho a ello; en este sentido, aquí está en juego un importante problema de comunicación (67). Por lo tanto, llevar a cabo la evaluación de las necesidades es muy importante para lograr que la intervención alcance sus objetivos (68).

Esta noción también está acorde con la evaluación de las necesidades mediante la comprobación de los medios económicos (ingresos) o las necesidades descritas anteriormente (42). Carey et al. apuntaron que el universalismo proporcional debería poner en práctica un selectivismo positivo (es decir, evaluar las necesidades y no los medios económicos), en particular porque las anteriores experiencias de comprobación de los medios económicos, realizadas en países anglosajones, obtuvieron peores resultados en cuanto a equidad (69).

### Desafíos éticos y de evaluación

En algunos artículos se abordaron problemas éticos centrándose en algunos grupos poblacionales más necesitados (48,63,70–72). En este contexto, la estigmatización es uno de los problemas más observados y debe evitarse. Por ejemplo, los estudios cualitativos realizados con madres en el contexto de intervenciones de visitas domiciliarias mostraron que estas tenían un sentimiento de culpa, y a veces percibían una actitud crítica de los profesionales hacia ellas (73). Por último, Bekken y Dierckx destacaron la necesidad ética de investigar qué saben acerca de estos conceptos los trabajadores sociales y de salud (57,74).

Muchos expertos han observado dificultades para evaluar adecuadamente la reducción de las desigualdades en la salud y, en mayor medida, la reducción del gradiente social (20,15,27,75,59). Los análisis a menudo se limitan al nivel consolidado, y evalúan más los resultados que los propósitos redistributivos (14). La ciudad de Richmond ha propuesto una solución, a saber, elaborar indicadores cuantitativos para evaluar la repercusión de su programa de la salud en todas las políticas y sugerir a su consejo municipal metas de desempeño por alcanzar, expresadas a través de indicadores como el porcentaje de ciudadanos que no sufren racismo, el porcentaje de empleados públicos de la ciudad que son mujeres o pertenecen a minorías, etc. (65).

## DISCUSIÓN

El objetivo principal de nuestra revisión sistemática exploratoria fue mapear la bibliografía científica disponible sobre el universalismo proporcional y sus conceptos conexos. Describimos los fundamentos teóricos que subyacen al universalismo proporcional y teorías principalmente sociales como la paradoja de la redistribución o el direccionamiento en el marco del universalismo propuesto en los últimos cuarenta años. Los análisis de los detalles de estas teorías permitieron comprender mejor las cuestiones prácticas planteadas por las aplicaciones del universalismo proporcional (o sus conceptos conexos). ¿Cómo ejecutar intervenciones proporcionales? ¿Qué umbral hay que aplicar para determinar el nivel de desventaja? ¿Qué indicador debe usarse para definir el nivel de desventaja? ¿Cómo demostrar la reducción eficaz del gradiente social de la salud?

El análisis de las aplicaciones del universalismo proporcional proporcionado en esta revisión respondió a algunas de estas preguntas, pero algunas de ellas son retos metodológicos que habrá que abordar en ulteriores investigaciones.

La definición precisa y práctica del principio no parece estar consensuada y puede dar lugar a diferentes interpretaciones (45). En efecto, las intervenciones que se refieren al universalismo proporcional fueron escasas y no siempre cumplían plenamente el principio: solo estaban orientadas a un grupo específico (no eran universales) o los autores no tuvieron en cuenta la reducción de las desigualdades como un resultado.

Todas las cuestiones planteadas por los especialistas en ciencias humanas y sociales con respecto a las teorías del universalismo o el direccionamiento pueden aplicarse al contexto de la salud pública y a las aplicaciones del universalismo proporcional. En la descripción de las aplicaciones del universalismo proporcional, se han destacado cuestiones más específicas relacionadas con el diseño y evaluación de intervenciones relacionadas con el universalismo proporcional (figura 2 y cuadro 3).

En particular, la atribución proporcional de una política planteó muchas cuestiones prácticas (determinación de necesidades, proporcionalidad exacta al gradiente, etc.), pero no debe olvidarse el aspecto universal. Efectivamente, cuando se plantea cómo reducir las desigualdades en la salud, desde el principio se abordan con demasiada frecuencia los grupos poblacionales que corren mayor riesgo (56). También debería ser muy interesante examinar más profundamente los conocimientos y percepciones de los profesionales de primera línea y los ciudadanos sobre estas cuestiones (74). Se está gestando una vasta bibliografía para comprender sus conocimientos e involucrarlos activamente en la reducción de las desigualdades sociales (76–79).

### Puntos fuertes y limitaciones

Hasta donde sabemos, esta es la primera revisión bibliográfica centrada en el universalismo proporcional. La presente revisión sistemática exploratoria es fruto de las recomendaciones anteriores, y la doble selección llevada a cabo por dos autores redujo el riesgo de clasificaciones erróneas. Solo elegimos algunas palabras clave sinónimas de universalismo proporcional, lo que no garantiza la exhaustividad. Sin embargo, nuestro objetivo no era ser exhaustivos, sino identificar a los autores que se reconocen en este término desde una perspectiva de salud pública. Por otro lado, la mayoría de los artículos incluidos se referían a países europeos (n = 39) donde prevalece el estado del bienestar y se originó el universalismo proporcional. Incluso realizando búsquedas con conceptos conexos, encontramos escasas intervenciones australianas, americanas o asiáticas que hicieran referencia al universalismo proporcional. Esto no significa que dichos conceptos no se usen, sino que los investigadores de esos países probablemente mencionen otros conceptos o no los citen con los sinónimos que usamos. Esto debe investigarse más a fondo. Asimismo, decidimos no centrarnos en la bibliografía gris, para recoger las opiniones de los investigadores sobre el universalismo proporcional. Sin embargo, una rápida investigación con la palabra clave “universalismo proporcional” obtiene pocas referencias a la aplicación y cita del principio del universalismo proporcional por parte de las autoridades locales de salud europeas (Marmot Cities, por ejemplo). Cabe señalar nuevamente que sería muy útil entrevistarlas y evaluar sus nociones sobre el universalismo proporcional.

### Factores determinantes ascendentes y descendentes

El término universalismo proporcional se propuso juntamente con seis objetivos de política: facilitar a todos los niños el mejor comienzo en la vida; permitir que todos los niños, jóvenes y adultos potencien al máximo sus capacidades y tengan control sobre su vida; crear empleo justo y un buen trabajo para todos; asegurar un nivel de vida saludable para todos; crear y desarrollar lugares y comunidades saludables y sostenibles; y mejorar el desempeño y los resultados de la prevención de la enfermedad (3). Por consiguiente, el universalismo proporcional se propuso como un medio de poner en práctica políticas ascendentes, procurando abordar las causas fundamentales de las desigualdades.

Desde esta perspectiva, los resultados de nuestra revisión muestran una realidad totalmente distinta: salvo en el caso de las intervenciones relativas a la crianza, la mayoría de los artículos se referían directamente a intervenciones de universalismo proporcional descritas que se centraban en los factores determinantes descendentes para mejorar el acceso a la prevención o atención (32,35,36,39).

Esto no necesariamente es contradictorio; todas esas intervenciones, tanto ascendentes como descendentes, pueden ser vistas como medidas complementarias que actúan a diferentes niveles y abordan los problemas en su origen y las consecuencias negativas de una falta de acción a un nivel más cercano.

Muchas intervenciones de salud pública incluidas en nuestra revisión han aplicado el universalismo proporcional para abordar los problemas relativos al acceso a la atención, o los factores determinantes descendentes. Esto también puede explicarse por el hecho de que las intervenciones de salud pública, cuando son eficaces, han demostrado ampliar las desigualdades en la salud (80).

Por el contrario, el número relativamente bajo de artículos médico-económicos encontrados en la revisión indica que los economistas, a pesar de estar muy familiarizados con las cuestiones relativas al universalismo y el direccionamiento, hacen mucho menos referencia al principio del universalismo proporcional. Sin embargo, los estudios que evalúan la eficacia y eficiencia de las políticas redistributivas para reducir las desigualdades en la salud son muy numerosos (81-83). En efecto, no es una tarea fácil distinguir el enfoque del universalismo proporcional de los enfoques más clásicamente “distributivos” que evalúan una intervención, un programa o una política en relación con objetivos distributivos (por ejemplo, conceder mayor importancia a aumentar la tasa de detección de poblaciones desfavorecidas porque presentan una tasa baja de detección). Habida cuenta de estos elementos, parece esencial iniciar un diálogo multidisciplinario para lograr un enfoque holístico del universalismo proporcional.

## Conclusiones

Esta revisión nos permitió mapear la bibliografía científica disponible sobre el universalismo proporcional y sus conceptos conexos. El principio del universalismo proporcional tiene su origen en teorías sociales: direccionamiento en el marco del universalismo, paradoja de la redistribución. Tal como destacan los autores que ejecutaron intervenciones de universalismo proporcional, su aplicación plantea muchos desafíos, desde el diseño hasta la evaluación. El análisis de las aplicaciones del universalismo proporcional proporcionadas en esta revisión respondió a algunos de ellos, pero en ulteriores investigaciones será necesario abordar los desafíos metodológicos restantes.

## Declaración.

Los autores son los únicos responsables de las opiniones expresadas en el texto, que no necesariamente reflejan la opinión o la política de la *RPSP/PAJPH* o la OPS.
